# Facility Death Review of Maternal and Neonatal Deaths in Bangladesh

**DOI:** 10.1371/journal.pone.0141902

**Published:** 2015-11-05

**Authors:** Animesh Biswas, Fazlur Rahman, Charli Eriksson, Abdul Halim, Koustuv Dalal

**Affiliations:** 1 Department of Public Health Science, School of Health and Medical Sciences, Örebro University, Örebro, Sweden; 2 Centre for Injury Prevention and Research, Bangladesh (CIPRB), Dhaka; BRAC, BANGLADESH

## Abstract

**Objectives:**

To explore the experiences, acceptance, and effects of conducting facility death review (FDR) of maternal and neonatal deaths and stillbirths at or below the district level in Bangladesh.

**Methods:**

This was a qualitative study with healthcare providers involved in FDRs. Two districts were studied: Thakurgaon district (a pilot district) and Jamalpur district (randomly selected from three follow-on study districts). Data were collected between January and November 2011. Data were collected from focus group discussions, in-depth interviews, and document review. Hospital administrators, obstetrics and gynecology consultants, and pediatric consultants and nurses employed in the same departments of the respective facilities participated in the study. Content and thematic analyses were performed.

**Results:**

FDR for maternal and neonatal deaths and stillbirths can be performed in upazila health complexes at sub-district and district hospital levels. Senior staff nurses took responsibility for notifying each death and conducting death reviews with the support of doctors. Doctors reviewed the FDRs to assign causes of death. Review meetings with doctors, nurses, and health managers at the upazila and district levels supported the preparation of remedial action plans based on FDR findings, and interventions were planned accordingly. There were excellent examples of improved quality of care at facilities as a result of FDR. FDR also identified gaps and challenges to overcome in the near future to improve maternal and newborn health.

**Discussion:**

FDR of maternal and neonatal deaths is feasible in district and upazila health facilities. FDR not only identifies the medical causes of a maternal or neonatal death but also explores remediable gaps and challenges in the facility. FDR creates an enabled environment in the facility to explore medical causes of deaths, including the gaps and challenges that influence mortality. FDRs mobilize health managers at upazila and district levels to forward plan and improve healthcare delivery.

## Introduction

Reducing maternal and newborn deaths is an integral part of the global agenda to achieve Millennium Development Goals (MDGs) 4 and 5 by 2015. Recent data have shown that the majority of maternal and neonatal deaths occur in developing countries: as many as 95% of total maternal and child deaths occur in 75 low- and middle-income countries [1]. Countries within Asia are at particularly high risk. Bangladesh has made encouraging progress in reducing maternal and neonatal mortality over the past two decades; since 1990, maternal mortality has fallen by two-thirds [2] and neonatal mortality has declined by over 50 per cent [3]. In 2010, Bangladesh received a United Nations award for its achievements in working towards attaining the MDGs, an acknowledgement of the great progress made in reducing maternal and neonatal mortality over the past two decades. Maternal mortality has reduced from 574 deaths per 100,000 live births in 1991 to 194 deaths in 2011. Neonatal mortality declined from 52 to 37 deaths per 1000 live births (38%) between 1989 and 2009 [4]. Recently, the Bangladesh Health and Demographic Survey showed that neonatal mortality had further reduced to 28 per 1000 live births [5]. Although the Government of Bangladesh is determined to maintain this progress, institutional delivery uptake is still poor, occurring in only 28.8% of cases [6, 7]. Recent data in Bangladesh from the maternal death review highlighted that 47.8% of maternal deaths occurred in facilities [8] and that in most cases the deaths could have been prevented.

Facility death review (FDR) is an important method for exploring gaps and delays in the system by examining the causes of death at health facilities, the aim being to improve the quality of care in hospitals [9]. In 2004, the World Health Organisation (WHO) formalized the FDR system for maternal deaths [10]. The WHO developed the Commission on Information and Accountability for Women’s and Children’s Health (COIA) Secretariat in Bangladesh to strengthen the death review system and also implement the WHO Maternal Death Surveillance and Response (MDSR) program [11].

The death audit approach is one of the most effective methods for improving health service performance to reduce maternal and perinatal deaths in healthcare facilities [12,13]. FDR is practised for maternal deaths in a number of countries [9, 14–22]. However, there is a paucity of comprehensive reviews of facility deaths in Bangladesh [20]. Bangladesh introduced a maternal and neonatal death review system for both community and facility maternal and neonatal deaths (including stillbirths) in 2010 [23]. This study explores FDRs of maternal and neonatal deaths and stillbirths and the experiences, effects, and lessons learnt to date in two districts in Bangladesh.

## Methods

This was a qualitative study performed in the Thakurgaon and Jamalpur districts of Bangladesh. The FDR (maternal and neonatal death review; MNDR) was initially piloted in the Thakurgaon district of Bangladesh in 2010. The district population is around 1.4 million and is situated in the northern part of the country. After a one-year MNDR pilot study, it was extended to another three districts in Bangladesh including Narail, Jamalpur, and Moulvibazar. For the purpose of meeting the aims of this study, we chose Thakurgaon as a pilot district, and Jamalpur district was randomly selected from the other three districts; Jamalpur covers a population of around 2.2 million people. From each district, three upazilas (sub-districts) were randomly selected for study. In both districts, FDRs were examined in the district hospitals, the maternal and child welfare centers (MCWCs), and six upazila health complexes (UHCs): 16 facilities were studied in total. The MNDR system was implemented by the Directorate General of Health Services (DGHS) in collaboration with the Directorate General of Family Planning (DGFP) under the auspices of the Ministry of Health and Family Welfare in Bangladesh. The MNDR development process is described elsewhere [8, 23–24].

The FDR process and its acceptance were assessed at the end of initial piloting during January 2011 in Thakurgaon district. To explore the effect of FDR, including the lessons learnt and the challenges highlighted, data were collected from both the pilot district (Thakurgaon) and the second district (Jamalpur) in November 2011.

Focus group discussions (FGDs) and in-depth interviews (IDIs) were conducted with facility doctors and nurses. Upazila and district health managers in both districts were also interviewed. Documents related to the development of FDR were reviewed, which included meeting minutes, different reviewed national and international tools, record notes, training guidelines, and manuals. These were collected so that each document could be thoroughly reviewed to understand the FDR development process. All the different document types were useful for uncovering their meaning, developing understanding, and discovering insights with respect to the research objectives [25]. This was also used as easily available, efficient, and less obstructive supplementary data with broad coverage and precision. All documents were examined twice to help understand the development process, after which content analysis was performed using a method appropriate for text analysis [26].

Thakurgaon district hospital, MCWC, and three UHCs were selected to identify the acceptability of FDRs. IDIs were conducted with doctors and nurses, one from each of the UHCs. Moreover, upazila health and family planning officers of those upazilas were also interviewed. In total, 13 IDIs were performed. Moreover, an FDG was conducted with the district hospital facility death review team, which consisted of doctors and nurses working in obstetrics and gynecology and pediatrics departments in Thakurgaon. There was a total of 11 participants: five doctors and six nurses (Table 1).

**Table 1:** Qualitative methods used in January 2011 in Thakurgaon district.

Methods	Responders	Thakurgaon	Upazila / district
In-depth interview	Doctors	4	One from each UHC and one from district hospital
In-depth interview	Senior Staff Nurses	4	One from each UHC and one from district hospital
In-depth interview	UHFPO	3	One from each UHC
In-depth interview	Civil Surgeon & Deputy Director of Family Planning	2	At district
Focus group discussion	Doctor and nurses	1 (Doctors = 5, Nurses = 6)	District hospital

To identify the effects of FDR, including lessons learnt and challenges discovered, three UHCs from Jamalpur district and in Thakurgaon were randomly selected; data were collected from the same three upazilas where acceptability data collection was performed. In addition to the UHCs, information was also collected from Thakurgaon and Jamalpur district hospitals.

A total of 12 IDIs were conducted with doctors and nurses primarily and actively involved in the facility death review process in both districts. In addition, IDIs were conducted with upazila health and family planning officers (Table 2).

**Table 2:** Qualitative methods used in November 2011 at Thakurgaon and Jamalpur district.

Methods	Responders	Thakurgaon	Jamalpur
In-depth interview	Doctors	3	3
In-depth interview	Senior Staff Nurses	3	3
In-depth interview	UHFPO	3	3
In-depth interview	Civil Surgeon	1	1
In-depth interview	Deputy Director of Family Planning	1	1

Each IDI was carried out with the doctors and nurses in their working facilities and took 20–30 minutes. Civil surgeons were interviewed at the district hospital, and deputy directors of family planning were interviewed at the district maternal and child welfare centers.

The anthropologists designated as research officers were formally trained prior to conducting the interviews. Pre-testing was performed before the study to finalize the guidelines. FDG and IDI participants provided informed verbal consent before the discussions and interviews. Informed consent was taken from each respondent before start of interviews, with tape recording only used after prior permission from respondents. Research officers made formal appointments with district and upazila health managers before the interviews. Since it was difficult to obtain written consent from each participant (doctors and nurses) during hospital working hours, both the investigators and participants were comfortable providing verbal consent before initiation of interviews in this setting. The ethics committee approved the taking of verbal consent.

Transcripts were prepared from audio records and handwritten notes in the local Bengali language and then translated to English. The research investigator was responsible for observing and monitoring the interviews in the field and also participated in the FGD sessions. Based on the findings, the investigator provided feedback to ensure data quality. Ten percent of transcripts were reviewed and checked for the quality of transcription from recorders; all transcripts were in the acceptable range for analysis. Data were first examined superficially to review its content, prior to thorough reading, examination, and interpretation. Open coding followed by selective coding was performed systematically by constantly moving backwards and forwards in the entire dataset and collecting relevant codes. Codes were then organized into different potential themes. The analysis was completed using a manual thematic approach, which is a flexible method for qualitative data analysis.

### Ethical Statement

The Ethical Review Committee, Centre for Injury Prevention and Research, Bangladesh reviewed the study protocol.

## Results

### Reviewed Documents

The facility death review instrument was initially developed prior to piloting in 2010 by reviewing the death review forms used in tertiary medical college hospitals and district hospitals and some other tools used by the Obstetrical and Gynecological Society of Bangladesh, Lamb Hospital. The WHO death audit form was also reviewed [10]. The DGHS and DGFP jointly organized a number of technical workshops and meetings in the presence of different government stakeholders and professional experts including obstetricians and gynecologists, neonatologists, and public health specialists. Developmental partners developed a simpler version of FDR that was effective for collecting data from district and lesser facilities.

**Fig 1:**
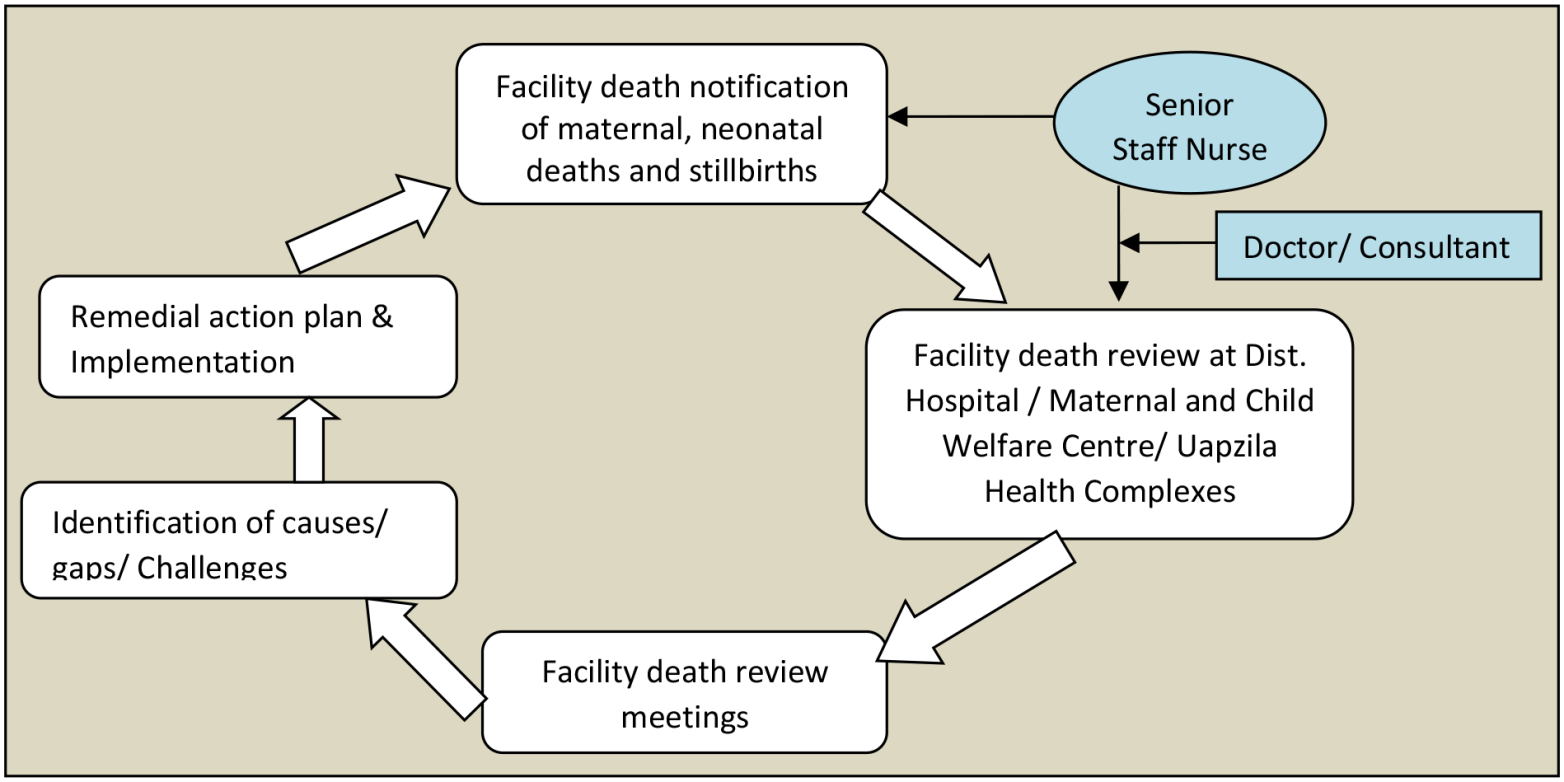
Facility death review in MNDR.

FDR involved a participatory and non-blaming process of reviewing any maternal deaths, neonatal deaths, and stillbirths in each facility. In the MNDR system, FDRs took place in the district hospital, maternal and child welfare centers, and UHCs. Senior staff nurses were assigned to perform facility death notifications of maternal and neonatal deaths and stillbirths in the district hospitals and UHCs, while family welfare visitors conducted FDRs in the district maternal and child welfare centers. This was followed by reviewing the deaths using existing records and recall by visiting the health care providers in the facility who treated or managed the patient prior to death. Nurses sought support from consultants or trainee doctors to assign causes of death if not recorded. Facilities planned to conduct each FDR immediately after the death was reported at the facilities. Review meetings for analyzing the causes of deaths to prepare corrective action plans took place quarterly in the district and in upazilas (Fig 1).

Hospital administrators or managers, consultants in obstetrics and gynecology, pediatrics, and anesthesia, and nurses involved in the FDR process participated in death review meetings. In each facility, a death review committee was formed to review the deaths as per national agreed guidelines.

The review meetings analyzed relevant events, factors, and delays that occurred in relation to service provision at the facility. The process aimed to identify all remediable factors to prevent recurrence of these types of deaths. The FDRs maintained the highest possible level of confidentiality and ethics. All information and data remained confidential, and anonymous records were preserved at the focal point in UHCs or district hospitals. The information was not publicly published or used to punish or appraise anybody.

### Acceptance of Facility Death Review

After piloting FDR in Thakurgaon district, it was found that healthcare providers at the district hospital and UHCs actively participated in the FDR process. Nurses notified each of the maternal and neonatal deaths and stillbirths at the facility using a structured death notification form. This was followed by FDRs by senior staff nurses with the support of medical staff. The system encouraged healthcare providers to report each facility death in the system followed by ensuring death reviews and analyzing causes of death.

The participants mentioned that FDR was practical and could be conducted in primary and secondary hospitals to determine the causes of maternal and neonatal deaths. Many participants mentioned that it was possible to understand and know the different gaps and challenges present in the facility including in human resources, logistics, or equipment. The interviewed doctors mentioned that FDR was a good quality of care indicator in the hospital, and felt that it was valuable and necessary to improve the quality of maternal and neonatal health services at the district level and below.

One of the doctors reported:

*“This is very good for our country context where we have adopted an easy and practical facility death review tool to get information on why the mothers and children are dying*. *We can find out the causes…we can build awareness*. *This is an excellent idea…”*

Health providers readily welcomed FDR. After conducting the death review at a facility, each cause of death was identified and discussed among providers. This helped them to understand the gaps and errors behind a death, including the role of facility delays. FDR provided them with the tools to rectify and take corrective measures to prevent deaths in the future.

During interview, one of the senior staff nurses mentioned:

*“It’s an opportunity for us to know why a death occurred and how it can be prevented in the future*. *I am pleased to engage in FDR*, *I try to collect all information provided by the tool*.*”*

In an FGD, another senior staff nurse stated:

*“It’s my responsibility to implement the death review and my colleagues help me to fill in the form*. *Many of the questions require support from others who are familiar with the case so I collect information from them*.

One of upazila health and family planning officers (UHFPO) of an upazila said:

*“Our upazila health complex has identified FDR as an important component in health service improvement*, *we are trying to work together even if we have a shortage of human resources”*.

The civil surgeon of Thakurgaon district stated:

*“FDR is excellent tool to find out the causes of deaths and explore the gaps and challenges in the hospital in order to improve quality of care at the facility level*,*”*

while the deputy director of family planning of Thakurgaon district said:

*“We had a tool supplied by the government to use if any death occurred at the maternal and child welfare centre*. *We were not using it properly*. *After the FDR process was initiated under MNDR*, *we are now performing FDR for each death*.*”*

### Effect of FDR at the Facility

Facility death review of maternal and neonatal deaths, including stillbirths, is a means for healthcare providers to look at the gaps and challenges in the facility where a death occurred. Periodic meetings were conducted at facilities by a combination of doctors and nurses working in maternity or neonatal wards to discuss the deaths that had occurred each month and for which corrective measures could be implemented (Box 1). Some of the gaps identified and mentioned by the participants were explored following facility death review. The data showed that record keeping at the district hospital and UHCs was poor, especially during admission when emergency patients’ details were not adequately noted. In cases where a death occurred at a facility and was not recorded correctly, possible community delays could not be established. In both district hospitals, mothers were mainly dying due to post-partum hemorrhage. FDR meetings identified that if blood supplies were available at the time of emergency then mothers’ lives could be saved. Birth asphyxia was the cause of death in the majority of neonates in neonatal wards. Discussions were held to ensure the availability of oxygen, Ambu bag ventilation, functioning incubators, and clean wards. Positive outcomes resulting from FDR findings that potentially saved mothers’ lives in Thakurgaon are shown in Box 2.

**Box 1. Key effects of FDR at facilities.**

- Created a platform to discuss deaths on a periodic basis.
- Discovered the gaps and challenges related to deaths.
- Corrective measures were taken and used as good examples after FDR.
- Acted as an indicator of quality of care at the facility.
- Strengthened responsibilities of the healthcare providers.
- Documentation process of patient records enriched.
- Improved supervision and monitoring systems.

**Box 2. A case study of a mother’s life saved from facility death review initiatives.**

During this period, a 19-year-old mother came to Thakurgaon district hospital with severe post-partum bleeding complications. She had had a home delivery in a Ranishankoil upazila village by an untrained birth attendant, after which she had severe post-partum bleeding. She went to an upazila health complex and was immediately referred to the district hospital. The mother’s life was saved at the district hospital because of a recent blood donor group initiative based on post-partum bleeding maternal death cases of 2010. The mother was given 19 bags of whole blood from the blood donor list and the hospital used the donor network to help the mother survive. The mother was later able to feed her child and was discharged healthy.

During an IDI, one of the doctors in Thakurgaon said:

*“When we discussed why deaths occurred in our facility using our death review form*, *we discovered that much of the information couldn’t be retrieved because of poor documentation and record keeping*. *We took initiatives to overcome the situation*.*”*

One of the senior staff nurses working in Jamalpur reported in an IDI that:

*“Now we are watchful for each vulnerable patient*. *We write down what we have done for the patient who had died and the cause of death*. *This information is now noted in the treatment card as well as in the register*.*”*

One nurse in the Jamalpur district hospital said:

*“When I visit the wards I saw that in the neonatal ward the register book the treatment sheet was completely filled up*. *I had never witnessed this before and in the other wards these documents are not completed properly*. *In the neonatal ward*, *the papers are always updated*.*”*

A doctor working in Thakurgaon district hospital mentioned:

*“We come to know for the first time that 12 maternal deaths had occurred in the district hospital in 2010*, *mostly due to post-partum haemorrhage and the fact that blood couldn’t be provided at the right time*. *Facility death review discussed detailed findings and at the meeting it was decided to form a 24-hour blood donor and functional blood availability list*. *We are happy that now we have a long donor list and we can call people during an emergency*.*”*

An UHFPO of one of the UHCs in Jamalpur said:

*“I also have responsibility to supervise and monitor the death review process and if any gaps are identified and discussed in our team we try to solve it locally*.*”*

The civil surgeon of Thakurgaon district said:

*“Quality of care can be ensured from facility death review*. *Now we are discussing why a death occurred*, *which was not done before*. *That’s a good indicator; we are now aware of each of the deaths*.*”*

### Lessons Learnt and Challenges

FDR at the district and UHC level is important and necessary and helps to identify some of the key causes and factors associated with causes of death including deficiencies within facilities. FDR discussion between healthcare providers was useful for discussing the deaths that had occurred and any remedial action that could be undertaken based on available resources to strengthen the quality of care at the facility. FDR was also essential at this level to ensure a continuum of care for patients, which in itself improves the quality of services at the district level. A number of examples of how facility death review mobilized the health care providers to think of and act on how to improve facility care are shown below.

An UHFPO at one upazila in Jamalpur mentioned:

“*FDR is essential to us; we now have the scope to analyse a death and we also plan to prevent such a death in the future*.*”*

One of the nurses working in Thakurgaon said:

*“We are now conducting facility death notification and review and are staying close to the patient so we can easily collect data*.*”*

Another nurse from Jamalpur said:

*“FDR is possible to perform in upazila health complexes*, *and we are now much better than before at patient record keeping—our health complex is now aware of the need for this*,*”*

while another nurse said:

*“Immediately after a death occurs it is now our responsibility and part of our work to undertake the facility death notification and review*, *and we do not delay in collecting the information*.*”*

However, some challenges were highlighted during in-depth interviews. Thakurgaon district was found to be more confident in patient information record keeping than Jamalpur district. In many cases it was found that patient history was incomplete, including the patient’s address in the community. At the district hospital, the death review was sometimes delayed due to nurses’ priority involvement in patient management. Moreover, it was also found that the causes of deaths were not recorded properly in many cases and the ICD-10 classification was missing (Box 3).

**Box 3. Challenges in facility death review.**

- Limited information was written on admission forms.
- The complete address and patient profile was not recorded properly.
- Insufficient follow-up records.
- Investigations and findings were missed in many cases.
- Different treatment times were not properly recorded.
- Diagnosis at the time of admission and facility diagnosis were blank on many cases.
- Causes of death did not follow ICD-10.
- Inadequate human resources, including nurses and doctors, considering patient admitted to perform FDR.
- The lengthy recall period made it difficult to obtain missing information.

One UHFPO in Thakurgaon mentioned:

*“The most challenging aspect of FDR is to accurately record the medical treatment and past history*, *which helps to identify factors associated with death*. *This accurate record keeping needs to be improved*.*”*

Another UHFPO in Jamalpur said:

*“We are at an initial stage of doing facility death review*. *It will take another year to get outputs from the death review process”*.

The civil surgeon of Jamalpur district said:

*“We have a deficit of nurses in our district hospital and we are in huge trouble due to patient overload and struggle to ensure optimum care*. *That has caused significant delays in implementing FDR*. *We have to depend on the recall of memories to know about death cases*.*”*

The DDFP of Thakurgaon district said:

*“FDR helps us to strengthen patient documentation*, *at least in the maternal and child welfare center we are improving compared to 2010*.*”*

## Discussion

Here we show that FDR has been introduced in district hospitals, maternal and child welfare centers, and UHCs in Bangladesh for the review of maternal and neonatal deaths including stillbirths. A vigorous process has been undertaken to adopt the tools and guidelines from existing instruments in overseas countries to prepare a practical and achievable death review system for primary and secondary health care centers. The government has approved the FDR instruments, including the facility death notification form and the death review form for maternal and neonatal deaths and stillbirths and guidelines used in districts to review facility deaths. The government health system has clearly played a key role in the implementation of death review. FDR for each maternal and neonatal death is practicable and functional, even considering the present health infrastructure including human resources and available facilities.

FDR of maternal deaths has previously been undertaken in Ethiopia with a system engaging healthcare providers in ten district hospitals, where it was found to be a powerful tool to maintain accountability [27]. Another study in northern Nigeria analyzed the experiences of maternal death review in 18 facilities and explored the existing maternal death review system and its challenges [9]. A study in Senegal demonstrated that maternal death review at facilities was feasible, especially in low resources settings [21]. FIGO LOGIC initiatives have shown that FDR resulted in significant health improvements at the local level [22]. Maternal and perinatal death reviews have been conducted in Tanzanian facilities since 2006 and have identified avoidable factors and opportunities for improvement via confidential multidisciplinary team discussions led by healthcare providers and by reviewing patient records [28]. A Lebanese study mentioned that the FDR approach helped professionals to understand the causes and determinants of maternal mortality [29]. In Asia, the WHO initiated maternal and perinatal death review at facilities in Bangladesh, Sri Lanka, India, Nepal, Myanmar, Bhutan, the Maldives, Indonesia, Thailand, and Korea [30]. In Bangladesh, one study on FDR reported that death review at a medical college hospital and district hospital provided the opportunity to identify substandard care at these facilities [20].

Here we examined beyond death review numbers and explored gaps and challenges [10]. FDR meetings were used as a platform for in-depth discussion of deaths and to identify service gaps behind the deaths that had occurred in the facility. A study on FDR reported that discussions with the healthcare providers at the facilities identified lessons and steps to further improve service [31].

There is some evidence to demonstrate examples of good practice to improve quality of care at facilities using the FDR system. The FDR system is simple and easy to implement; this is particularly evident when examining a case study of a woman where blood was managed using a donor network established through a vigorous review of maternal deaths during 2010 in Thakurgaon district hospital. Similar successes were found in Orissa, India, where FDR findings that post-partum haemorrhage was a major cause of maternal death were used to good effect: eight districts set up blood banks and a blood storage unit to improve patient survival. In Rajasthan, India, an obstetrics helpline and referral transport system was established for mothers [32].

A number of challenges were identified during FDR implementation, the most notable being inadequate patient information being documented in hospital records. This made it difficult to assess the real causes and gaps or challenges. There was also a major obstacle in the lack of space or time for healthcare providers to perform FDR. Delays in completing the FDR resulted in the loss of some data due to the prolonged recall period, sometimes over a month, even though the national agreed level to complete an FDR is within 15 days of a death occurring. In many cases, the transfer of trained healthcare providers (nurses and doctors) from the hospital decreased the facility’s ability to conduct FDR. One study on maternal death review in Africa also reported that frequent turnover of programme managers and staff caused difficulty in maternal death review [14].

Other countries have reported similar findings, with FDR found to be challenging due to deficiencies in basic patient registration details, record keeping, and skilled human resources to conduct the death review [9, 16, 20–21, 25–26, 33–35].

### Limitations

This study has a number of limitations. We did not interview anyone from the family planning departments of maternal and child welfare centers and, in both centers, maternal and neonatal deaths were not notified during duty time, only stillbirths. Therefore, we interviewed the deputy director of family planning of the district family planning department. Moreover, while exploring FDR acceptability, we performed an FGD in Thakurgaon district hospital. However, we did not conduct any FGD in the latter part of data collection in September 2011. This was because organizing an FGD was difficult, with doctors and nurses mentioning that it created difficulties in looking after patients. Therefore, we performed only interviews.

## Conclusions

The lack of information in patient records and human resource constraints are key challenges to ensuring quality FDR. However, the successes noted are also evidence to suggest improvement in the quality of care at health facilities due to FDR. Our study shows that facility-based case reviews are simple, non-blaming, and can easily be performed within the existing health system. Although FDRs require intensive supervision, monitoring, and support to overcome obstacles, they also provide the opportunity to improve outcomes for mothers and newborns at health facilities in Bangladesh.
